# The Fe-S cluster biosynthesis in *Enterococcus faecium* is essential for anaerobic growth and gastrointestinal colonization

**DOI:** 10.1080/19490976.2024.2359665

**Published:** 2024-06-03

**Authors:** Linan Xu, Yajing Wu, Xiangpeng Yang, Xinxin Pang, Yansha Wu, Xingshuai Li, Xiayu Liu, Yuzhong Zhao, Lumin Yu, Peikun Wang, Bin Ye, Shijin Jiang, Junfei Ma, Xinglin Zhang

**Affiliations:** a College of Agriculture and Forestry, Linyi University, Linyi, China; b Department of Preventive Veterinary Medicine, College of Veterinary Medicine, Shandong Agricultural University, Tai’an, China; c Department of Food Science and Nutrition, Zhejiang University, Hangzhou, China; d State Key Laboratory of Microbial Technology, Shandong University, Qingdao, China; e Institute of Edible Fungi, Shanghai Academy of Agricultural Sciences, Shanghai, China

**Keywords:** *Enterococcus faecium*, Fe-S cluster biosynthesis, anaerobic conditions, gastrointestinal (GI) tract colonization

## Abstract

The facultative anaerobic Gram-positive bacterium *Enterococcus faecium* is a ubiquitous member of the human gut microbiota. However, it has gradually evolved into a pathogenic and multidrug resistant lineage that causes nosocomial infections. The establishment of high-level intestinal colonization by enterococci represents a critical step of infection. The majority of current research on *Enterococcus* has been conducted under aerobic conditions, while limited attention has been given to its physiological characteristics in anaerobic environments, which reflects its natural colonization niche in the gut. In this study, a high-density transposon mutant library containing 26,620 distinct insertion sites was constructed. Tn-seq analysis identified six genes that significantly contribute to growth under anaerobic conditions. Under anaerobic conditions, deletion of *sufB* (encoding Fe-S cluster assembly protein B) results in more extensive and significant impairments on carbohydrate metabolism compared to aerobic conditions. Consistently, the pathways involved in this utilization-restricted carbohydrates were mostly expressed at significantly lower levels in mutant compared to wild-type under anaerobic conditions. Moreover, deletion of *sufB* or *pflA* (encoding pyruvate formate lyase-activating protein A) led to failure of gastrointestinal colonization in mice. These findings contribute to our understanding of the mechanisms by which *E. faecium* maintains proliferation under anaerobic conditions and establishes colonization in the gut.

## 1. Introduction

*E. faecium* is a Gram-positive bacterium and a member of the normal intestinal flora of animals.^1^ Additionally, there is substantiated evidence suggesting the presence of enterococci in plants, insects,^2,3^ water, soil, and mountainous regions.^4^ *Enterococcus* species exhibit adaptability to both aerobic natural habitats and anaerobic intestinal environments. This bacterium exhibits an exceptional capacity to endure adverse environmental circumstances, such as elevated temperatures and heightened salt concentrations.^5^ Moreover, *Enterococcus* species exhibit exceptional resistance to various chemical disinfectants, such as chlorine, glutaraldehyde, and alcohol.^6^ These distinct characteristics contribute to the persistence of *E. faecium* within the modern hospital environment and highlight its ability to adapt during long-term co-evolutionary processes. Over the past three decades, *E. faecium*, *Staphylococcus aureus*, *Klebsiella pneumoniae*, *Acinetobacter baumanii*, *Pseudomonas aeruginosa*, and *Enterobacter*, which are collectively known as ESKAPE, have been identified by the American Society for Infectious Diseases as significant causes of treatment challenges and mortality worldwide.^7^ *E. faecium* has emerged as a multidrug-resistant pathogen that is specifically associated with healthcare settings.^8^

The establishment of a heightened level of enterococcal colonization within the intestines is a critical stage in a process that has the potential to result in nosocomial infections.^9^ The capacity of bacteria to adapt to stress within the intestinal tract is crucial for survival and successful colonization. Throughout the gastrointestinal phase, intestinal bacteria confront the host’s formidable physiochemical defenses, which include the acidic pH of the stomach, heightened osmolarity and bile salts in the upper small intestine, as well as anaerobic conditions prevailing within the intestine.^10^ The gut is characterized by anaerobic conditions, resulting in the predominance of anaerobic microorganisms in the intestinal microbiota of mammals.^11^ The proportion of strict anaerobes in the gut increases progressively from the proximal to distal regions, constituting 99% of bacterial species within the colon.^12^ The oxygen partial pressure in the colonic mucosa has been observed to be less than 25% of atmospheric oxygen concentration.^13^ Despite being less abundant, facultative anaerobes such as *Escherichia coli* and *Enterococcus* are extensively present in the mammalian intestinal tract.^14^ These organisms can survive under both anaerobic and aerobic conditions, and the concentration of oxygen plays a regulatory role in their growth and metabolism.^15^ For instance, oxygen levels in the intestinal wall play a significant role in the virulence of pathogenic bacterium *Shigella*.^16^ Despite the vital importance of oxygen levels in the colonization and virulence of intestinal microbes, limited research has been conducted in this particular field.

The microbiota could play a crucial role in modifying the gut environment, indirectly enhancing resistance against enteric pathogens and pathobionts.^17^ A notable example of this phenomenon is the ability of symbiotic bacteria to establish and maintain intestinal hypoxia, which limits the proliferation of facultative anaerobic pathogens.^18^ Consequently, facultative anaerobes in symbiosis can competitively acquire or sequester residual oxygen within the gut, further restricting pathogen colonization.^16^ Furthermore, it has been observed that other symbionts can compete for anaerobic respiration substrates in the absence of oxygen.^19–21^ Previous studies have investigated the impact of oxygen limitation on the abundance of Enterobacteriaceae in the gastrointestinal tract, revealing that a scarcity of respiratory electron acceptors limits the growth of Enterobacteriaceae within a balanced gut-associated microbial community^22^ and gastrointestinal colonization. As a facultative anaerobic bacterium, *E. faecium* grows most rapidly when respiring oxygen and switches to anaerobic respiration in the absence of oxygen.^23^ To thrive, *Enterococcus* must undergo adaptations in an environment lacking oxygen. However, the specific genes involved in this adaptive process and their respective functions have not been extensively documented.

Transposon sequencing (Tn-seq) is a bacterial functional genomics research technique that combines transposon mutagenesis with high-throughput sequencing.^24^ Tn-seq has emerged as a genome-wide technique that establishes a direct association between phenotype and genotype.^25^ This technique facilitates the simultaneous pooling of millions of diverse mutant strains, enabling high-throughput identification and correlation analysis of gene function. Additionally, it allows for quantitative monitoring of variations in the relative abundance of mutants acquired during pre- and post-tests. Through Tn-seq, we can comprehensively identify conditionally essential genes in bacteria within specific growth environments and elucidate the functional roles of bacterial genes at a genome-wide scale.^26–29^

In this study, a high-density transposon library was constructed for *E. faecium* E980, allowing for comprehensive identification of genes associated with anaerobic conditions through Tn-seq analysis. As a result, *suf* gene cluster was identified. This gene cluster is widely regarded as the oldest known pathway for the biogenesis of iron-sulfur (Fe-S) cluster and is supposed to be involved in early anaerobic life forms.^30^ These Fe-S cluster play crucial roles as essential metal cofactors in various vital biological processes, including respiration, photosynthesis, nitrogen fixation, and DNA repair.^31,32^ The process of Fe-S biogenesis involves several steps: SufS facilitates mobilization of sulfide (in the form of persulfide) from L-cysteine, SufD acquires iron, and SufU transfers the obtained sulfur to the target scaffold proteins (SufB and SufC),^33^ as shown in Figure 3b. Previous studies have also reported the mechanism of Fe-S cluster generation by Suf system in *Enterococcus*,^34–36^ but the association of Fe-S cluster with bacterial growth characteristics and host interaction has not been described. In the present study, *suf* gene cluster of *E. faecium* was characterized, and Fe-S synthesis pathway encoded by *suf* genes was shown to play a significant role in anaerobic growth and gastrointestinal colonization of *E. faecium.*

## 2. Materials and methods

### 2.1. Bacterial strains, media, and growth conditions

Bacterial strains and plasmids used in this study are listed in Table S1. All strains were grown at 37°C in brain heart infusion (BHI) broth, and antibiotics (Solarbio) were used at the following concentrations: gentamicin, 200 µg/mL; spectinomycin, 100 µg/mL.

### 2.2. Construction of a mariner transposon mutant library in *E.* *faecium*

To create a high-density transposon mutant library, the temperature-sensitive plasmid pGPA2^37^ was electroporated into *E. faecium* E980. The mutant library was generated according to previously described methods.^38^ In brief, E980 containing plasmid were grown in BHI broth supplemented with chloramphenicol at 30°C overnight, after which cultures (200 μL) were added to BHI broth supplemented with gentamicin (25 μg/mL) and nisin (25 ng/mL) overnight. Then, cultures were incubated in BHI broth without antibiotics at 37°C for two successive passages. Subsequently, cultures were stored at −80°C in 50% (v/v) glycerol.

### 2.3. Tn-seq analysis of conditionally essential genes involved in anaerobic conditions

Tn-seq, a high-throughput tool for the functional genomic study of pathogens, has previously been described in detail.^39^ In this study, we used this technique to perform genome-wide identification of conditionally essential genes of *E. faecium* under anaerobic treatment. The procedure was similar to that described previously.^40^ *E. faecium* mariner-based transposon mutant library was grown in BHI broth supplemented with 25 µg/mL gentamycin to the stationary growth phase.

Subsequently, cultures were inoculated anaerobically at a concentration of 1 × 10^7^ CFUs/mL in BHI broth at 37°C for 10–12 h in an anaerobic incubator. Similarly, the control group was placed in a warm air incubator at 37°C for 10–12 h. Then, bacteria were collected immediately by centrifugation, and a genomic DNA kit (Qiagen) was used for genomic DNA extraction. Sample libraries were prepared and analyzed by barcoded Tn-seq as described previously.^41^ Library preparation and sequencing were performed on the Illumina HiSeq PE150 platform (Personalbio, Shanghai, China), generating an average of 1 G high-quality sequencing reads per sample. This experiment was performed in triplicate.

### 2.4. Bioinformatics analysis of Tn-seq data

Tn-seq data analysis was performed as described previously.^42^ After Illumina sequencing, the raw reads were split into groups based on their barcodes using Fastp.^43^ Sixteen nucleotide fragments of each read, corresponding to E980 genomic sequence flanking transposon, were mapped to E980 genome using Bowtie 2.^44^ Then, Integrative Genomics Viewer (IGV) was used to sort and count results of the alignment. Next, the read 6 counts of each gene were normalized by the following formula: RPTAM = (number of reads mapped to a gene × 10^6^/(total mapped input reads in the sample × number of TA sites in this gene). Cyber-T^45^ (http://cybert.microarray.ics.uci.edu/) was used for statistical analysis of RPTAM values between different groups. A *p* value < .05 was considered to indicate statistical significance. Only genes with a significant and unique association with either sensitivity or tolerance were identified.

### 2.5. Phylogenetic and *in silico* analysis of *suf* gene cluster in *E.* *faecium* E980

The BLAST tool of the NCBI (https://www.ncbi.nlm.nih.gov/) was used for homology analysis of *suf* gene cluster.

The amino acid sequences of SufB (Fe-S cluster assembly protein B) and PflA (pyruvate formate lyase-activating protein A) in *E. faecium* E980 were submitted to structural prediction using the AlphaFold2 server.^46^ Structural alignment of SufB (or PflA) in *E. coli* and E980 was performed using the TM-align server^47^ for structural analysis. Sequence alignment was performed using the CLUSTALW server,^48^ and visualization was achieved using the ESPript 3.0 server.^49^

To explore which Fe-S cluster-containing proteins downstream were affected by impaired Suf system, two methods were performed. Firstly, the homologous Fe-S cluster-containing protein in *E. faecium* was analyzed based on the literature report of Fe-S cluster-containing protein in *E. coli*.^50^ Moreover, the UniProt database was used to directly search for Fe-S cluster-containing proteins in *E. faecium*. The identified proteins by above methods were subsequently mapped to *E. faecium* E980 genome. We employed these two methods to identify Fe-S cluster-containing proteins potentially implicated in *E. faecium* E980 strain.

### 2.6. Construction and complementation of *E.* *faecium* E980 mutant strains

Deletion of *sufB* was performed in E980 strain through the recombination system mediated by plasmid pWS3.^51^ A schematic diagram of the recombination system for the construction of gene knockout mutant in *E. faecium* is provided in Supplementary Materials (Figure S1).The primers used are listed in Table S1. Plasmid pWS3 was digested with Sma I, and PCR fragments (gene_up, gene_dn, *gm*) were ligated by using NovoRec plus One step PCR Cloning Kit (Novoprotein Scientific, Inc., Shanghai, China). Plasmid pWS3_gene_*gm* was transformed into E980 competent cells using the electroporation method. As shown in Figure S2, a transformant containing plasmid was grown overnight in BHI broth at 30°C supplemented with gentamicin. Cell cultures were then diluted 10,000-fold in prewarmed BHI broth and grown at 37°C overnight without antibiotics. Cells were then plated on BHI agar plates with gentamicin and incubated at 37°C. Colonies were then restreaked on BHI agar plates with spectinomycin and BHI agar plates with gentamicin, respectively. The gentamicin-resistant but spectinomycin-susceptible colonies were supposed to be marked deletion mutants. Thus, marked deletion mutants were obtained. Deletion of *pflA* was performed via the same method. All plasmid constructs and gene deletions were confirmed by DNA sequencing.

Genetic complementation of mutants was performed as previously described.^52^ To complement Δ*sufB::gm*, *sufB* gene with its promoter was cloned and inserted into pMSP3535 shuttle plasmid. PCR products were cloned and inserted into *Bam*H I site of pMSP3535 vector using NovoRec® plus One-step PCR Cloning Kit (Novoprotein), generating plasmid pMSP3535-*sufB*. Positive transformants were obtained as previously described. Recombinant plasmids were subsequently introduced into Δ*sufB::gm* host strains by electroporation, after which transformants were selected at 37°C on BHI plates supplemented with erythromycin and lincomycin. Other complement strains were constructed via the same method.

### 2.7. Determination of growth curves

A growth curve was generated for wild-type (WT), isogenic mutant, and complemented strains under both aerobic and anaerobic conditions. Anaerobic operation was conducted within a specialized anaerobic incubator. Strains were grown overnight in BHI. 50 μL of overnight culture was inoculated into 50 mL of solution. Cultures were incubated in an incubator at 37°C, and absorbance at 600 nm (OD_600_) was recorded every hour for 9 hours. Each experiment was carried out in triplicate.

### 2.8. Transcriptome comparison between WT and Δ*sufB::gm* strains under aerobic and anaerobic conditions

WT and Δ*sufB::gm* were incubated in BHI broth under aerobic and anaerobic conditions, respectively, for 6 hours. Bacterial cells were centrifuged at 10,000 rpm for 1 min and snap-frozen in liquid nitrogen. RNA extraction and quantification were performed as described in previous studies.^53^ Then, amplified and purified RNA-Seq libraries were sequenced on the NovaSeq 6000 platform (Illumina, San Diego, CA, USA), and 150-bp paired-end reads were generated. Differentially expressed gene (DEG) analysis and enrichment analysis (Gene Ontology [GO] analysis) were performed according to previous methods.^53^

### 2.9. qPCR validation of RNA-Seq experiments

cDNA was synthesized according to instructions of the PrimeScript RT Reagent Kit with gDNA Eraser (Takara, Beijing, China). Then, qPCR was conducted using TB Green® Premix Ex Taq™ (Tli RNaseH Plus) Kit (Thermo Fisher Scientific, Waltham, MA, USA) and Applied Biosystems™ QuantStudio™ 3 instrument (Thermo Fisher Scientific, Waltham, Massachusetts, USA). Transcript levels of assayed genes relative to *divIVA* were calculated using QuantStudioTM Design & Analysis Software 1.3.1 (Thermo Fisher Scientific, Waltham, MA, USA). Data analysis was performed using the 2 −ΔΔCt method. Three biological replicates were performed for each group.

### 2.10. Carbohydrates metabolism test

To assess the metabolic capacities of WT strain, its isogenic mutants, and complemented strain under both aerobic and anaerobic conditions in relation to various sugars, namely, glucose, trehalose, sucrose, raffinose, arabinose, mannitol, cellobiose, galactose, lactose, xylose, xylitol, fructose, L -rhamnose, maltose, sorbitol, inulin and mannose, 17 microbiochemical identification tubes (Haibo, China) were employed. A total of 1 × 10^8^ CFUs of WT strain, its isogenic mutants, and complemented strains were inoculated with 300 μL of microbiochemical identification reagent, BHI broth was used for overnight culture of all strains. The results are recorded at two-hour intervals within a 24-hour period, and at twelve-hour intervals between 24 and 48 hours.

### 2.11. Intestinal colonization in mice

Colonization of intestines of mice was conducted using *E. faecium* strains following a previously described method with certain modifications.^38,54,55^ Specifically, before antibiotic treatment, 10 BALB/c mice were subjected to environmental adaptation for one week. seven-week SPF mice were subjected to a 3-day decolonization period via the oral administration of four antibiotics once daily.^56^ Mice were allowed to drink water supplemented with four antibiotics (1 g/L) ad libitum for 7 days. *Enterococcus* in fecal pellets was not detected after antibiotic therapy. Mice were then inoculated with 1 × 10^8^ CFUs in 300 μL of phosphate-buffered saline (PBS), which was an equal ratio of WT and Δ*sufB::gm*; both were cultured separately overnight in BHI broth. Feeding was performed as previously described with contaminated food.^38,54,55^ Pfizer *Enterococcus* Selective Agar (PESA; These plates support the growth of both WT and mutants) or PESA supplemented with 5 μg/mL gentamicin was used to plate the diluted inoculums. The viable counts for mutants and WT were measured in feces samples at 1, 3, 5, and 7 days after colonization. We euthanized mice after they had colonized for 7 days. Samples were collected, weighed, and homogenized in 10 volumes of PBS after being detached from cecum and colon, as shown in Figure 5a. To determine viable counts, serial dilutions of homogenates were performed, and subsequent plating on PESA or PESA supplemented with 5 μg/mL gentamicin was performed. Cells were incubated at 37°C for 2 days; subsequently, colonies were counted, and identity of strains were confirmed via PCR. Δ*pflA::gm* was treated similarly. The enteric competition test of WT and Δ*sufB::gm* + *sufB* was the same as enteric competition test of WT and mutant strains in the preliminary treatment. Mice were then inoculated with 1 × 10^8^ CFUs in 300 μL of PBS, which was an equal ratio of WT and Δ*sufB::gm* + *sufB*; both were cultured separately overnight in BHI broth. The viable counts for Δ*sufB::gm* + *sufB* and WT were measured in feces samples at 3, 5, and 7 days post colonization. Δ*pflA::gm* was treated similarly. We conducted a paired two-tailed Student’s t test to determine whether differences in the log-transformed data between WT and mutant strains were statistically significant. A *p* value < .05 was considered to indicate statistical significance. The Animal Care and Use Committee of Linyi University (Linyi, China) approved all the experiments.

### 2.12. Statistical analysis

All analyses of significance were performed using one-way analysis of variance (ANOVA) and Duncan’s multiple range test (DMRT). In all the analysis, only when *p* < .05 the data were considered to be statistically significant. All tests were conducted in triplicate.

## 3. Results

### 3.1. Construction and evaluation of a high-density transposon mutation library in *E.* *faecium*

**Figure 1.**
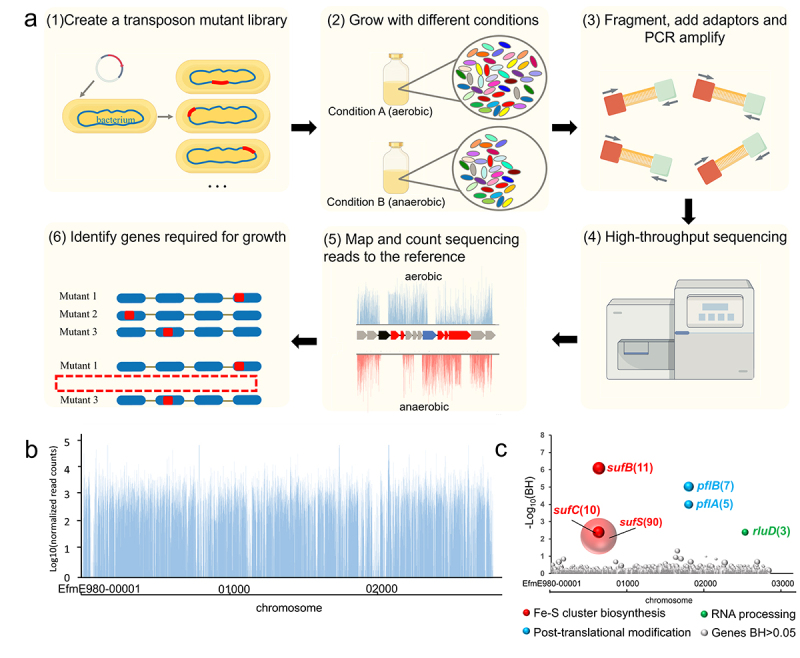
Tn-seq analysis for identification of functional genes under anaerobic conditions in *E. faecium*.

We constructed a mariner-based transposon insertion library in *E. faecium* E980, and the quality of this mutation library was evaluated by Tn-seq analysis. The key steps involved in Tn-seq are illustrated in Figure 1a. The sequencing results revealed that each sample exhibited a minimum of 15 million Tn-seq readings, with no discernible presence of biased transposon insertion sites. The transposon distribution exhibited a uniform and high-density pattern, as shown in Figure 1b, with a total of 26,620 distinct insertion sites identified across the entire genome (Table S2). Subsequently, a comprehensive analysis of Tn-seq data was conducted to discern the genetic determinants associated with anaerobic conditions.

(a) Schematic depiction of Tn-seq data. (b) In the control group, transposon insertion sites and insertion numbers at different sites and the presence of transposon insertions in 2702 genes within the E980 genome of *E. faecium* are indicated by blue lines. (c) Identification of functional genes implicated in resistance to anaerobic conditions through Tn-seq analysis. Distinct bubbles denote distinct genes, with bubble sizes corresponding to fold changes. Fold changes increase as the size of the bubble increases. The x-axis represents genomic position of genes on the chromosome, while y-axis represents outcome of statistical analysis of Tn-seq data. Genes exhibiting a significant change (BH < .05) in growth between anaerobic treatment and aerobic treatment groups are grouped by function and are labeled with different colors, and name and change in abundance between the control conditions and growth in anaerobic treatment group are indicated next to the bubbles in parentheses, while remaining genes are represented in gray.

### 3.2. Identification of genetic determinants involved in anaerobic growth by Tn-seq

To identify genes required for growth under anaerobic conditions in *E. faecium*, Tn-seq was performed to determine which mutants were selectively lost during anaerobic growth. The involvement of six genes (Benjamini – Hochberg corrected *p* value (BH) < .05) under anaerobic conditions was identified (Figure 1c and Table S2). Genes that had the most pronounced effect on the growth of *E. faecium* under anaerobic conditions included genes involved in Fe-S cluster biosynthesis (*sufB*, *sufC*, *sufS*), genes associated with post-translational modification (*rluD*) and genes involved in RNA processing (*pflA, pflB*) (Figure 1c).

### 3.3. Revealing essential genes of *E.* *faecium* E980 through Tn-seq analysis

By employing the EL-ARTISIT approach for identification of essential genes in E980 cells cultivated in BHI medium, a total of 631 essential genes were identified in Table S3, constituting approximately 21.25% of the entire gene repertoire. Essential genes identified in this study were submitted to GO and KEGG functional enrichment analysis, which provided insights into biological processes that are crucial for survival of *E. faecium*.

**Figure 2.**
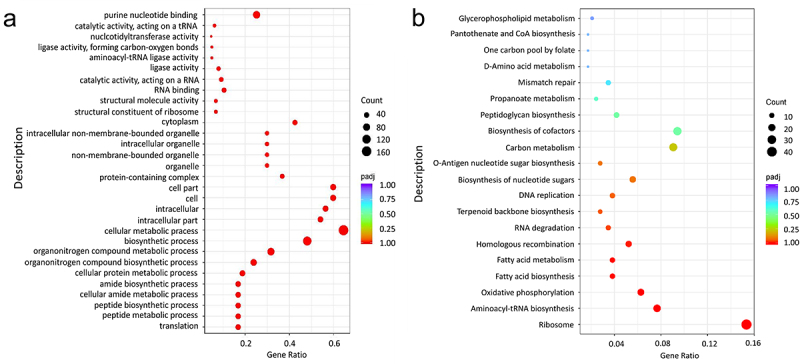
Essential gene of GO (a) and KEGG (b) enrichment analysis.

According to GO enrichment analysis (Figure 2a), a P_adj_ (corrected *p* value) < .05 indicated statistically significant enrichment in the GO functional terms. The set of essential genes exhibited significant enrichment in 88 biological process terms, 135 molecular function terms, and 13 cell component terms. The functions of essential genes are predominantly involved in translation, cell metabolism, and biosynthesis. These genes encompass conserved multi-component pathways essential for fundamental metabolic and structural functions in bacteria.

The enriched biological pathways are shown on the y-axis, and Gene Ratio was defined as the ratio of the number of DEGs annotated to GO term or on KEGG pathway to the total number of genes on the x-axis.

The KEGG pathway analysis is illustrated in Figure 2b. Essential genes exhibited enrichment in 48 biological metabolic pathways, with significant enrichment observed in 8 specific pathways, namely, ribosome pathway, aminoacyl-tRNA biosynthesis pathway, oxidative phosphorylation pathway, fatty acid synthesis pathway, fatty acid metabolism pathway, homologous recombination pathway, RNA degradation pathway, and terpenoid skeleton biosynthesis pathway. The enrichment of ribosome pathway was the most significant, with a notable abundance of enriched genes.

### 3.4. Phylogenetic and in silico analysis of suf gene cluster in *E.* *faecium*

**Figure 3.**
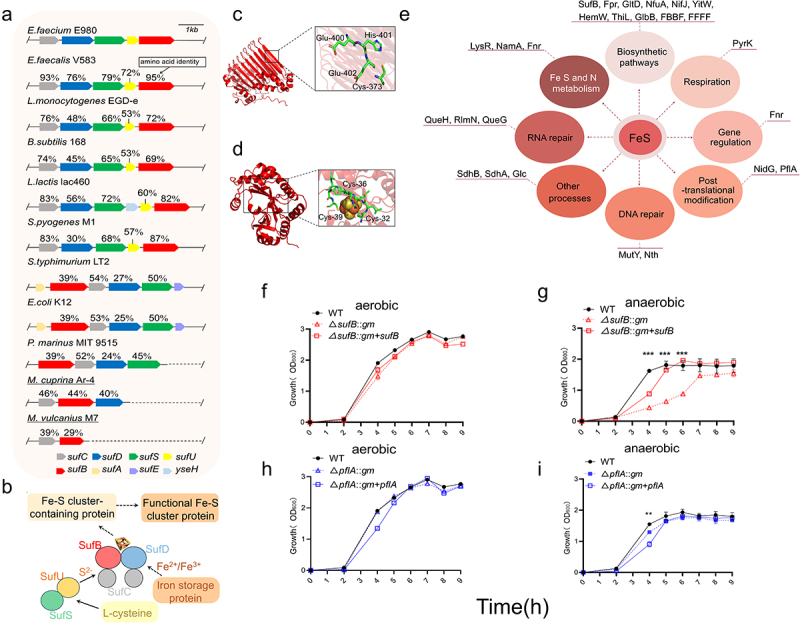
Phylogenetic and *in silico* analysis of Suf pathway.

A genome sequence analysis of *E. faecium* strain E980 was performed to identify *suf* gene cluster. The putative *suf* gene cluster included *sufC*, *sufD*, *sufS*, *sufU*, and *sufB* in E980 strain. To identify *suf* homologues in E980 genome, we searched for these homologues in 10 other species, namely, *Enterococcus faecalis*, *Listeria monocytogenes*, *Bacillus subtilis*, *Salmonella typhimurium*, *E. coli*, *Lactococcus lactis*, *Streptococcus pyogenes, Prochlorococcus marinus, Methanocaldococcus vulcanius*, and *Metallosphaera cuprina*. Among these bacteria, *Prochlorococcus marinus* is a bacterium with a simple structure; *Methanocaldococcus vulcanius* and *Metallosphaera cuprina* are archaea. The findings are illustrated in Figure 3a. The proteins encoded by this gene cluster are homologous to Suf proteins of *E. faecalis* V583 (amino acid identity: 76%–95%), *B. subtilis* 168 (45%–74%), *L. monocytogenes* EGD-e (48%–76%), *S. typh-imurium* LT2 (27%–54%), *E. coli* K12 (25%–53%), *L. lactis* lac460 (56%–83%) and *S. pyogenes* M1 (30%–87%). *P. marinus* MIT 9515 (24%–52%), *M. cuprina* Ar-4 (40%–46%), and *M. vulcanius* M7 (29%–39%) were used. These results indicate that *suf* gene cluster are widely distributed across a diverse range of species.

(a) Phylogenetic analysis of *suf* gene cluster of *E. faecium* E980. The *sufA*, *sufB*, *sufC*, *sufD*, *sufS*, and *sufU* genes are color-coded to reflect their homology in different organisms. (b). Illustrations of Suf pathway for Fe-S cluster biogenesis in bacteria. Solid arrows denote functional steps that are amply corroborated by *in vitro* and *in vivo* data. The dashed arrows represent steps that have yet to be comprehensively characterized. The depicted surface representation structures include SufB (red), SufC (gray), SufD (blue), the SufS homodimer (green), and SufU (yellow). The putative Fe-S cluster binding sites of SufB (c) and PflA (d) in *E. faecium*. The specific residues needed to ligate Fe-S cluster are illustrated as sticks. (e) *In silico* analysis of Fe-S cluster-containing proteins in *E. faecium* E980 (This image was created in part By Figuredraw). Growth curves of *E. faecium* under both aerobic and anaerobic conditions. Growth curves of WT (black, solid line), Δ*sufB::gm* (red, dotted line), and Δ*sufB::gm* + *sufB* (red solid line) under both aerobic (f) and anaerobic (g) conditions. Similarly, growth curves of WT (black, solid line), Δ*pflA::gm* (blue, dotted line), and Δ*pflA::gm* + *pflA* (blue solid line) under both aerobic (h) and anaerobic (i) conditions were generated. The growth curves represent the average data from three independent experiments. Asterisks indicate significant differences between WT and mutant strains according to Student’s t test: *** *P* ≤ .001, ** *P* < .05.

The predicted structure of E980 SufB protein exhibited a topological arrangement highly similar to that of SufB protein in *E. coli* (Figure S3a; TM-Score = 0.92,RMSD = 2.01). Additionally, the crucial functional sites 373C, 400E, 401 H, and 402E exhibited significant conservation (Figure S3c). These sites are predicted to be associated with Fe-S cluster biosynthetic function of SufB in *E. coli*.^57^ Therefore, we hypothesized that these sites also play similar roles in Fe-S biosynthesis of E980 SufB. These sites are shown in E980 SufB structure (Figure 3c). Similarly, structural and sequence alignment analyses of PflA in E980 and *E. coli* were performed (Figure S3b, D; M-Score = 0.95;RMSD = 1.73), and Fe-S cluster modification site^58^ of PflA was identified in *E. coli*. The potential Fe-S cluster modification sites of PflA in E980 were identified at positions 32C, 36C, and 39C (Figure S3d; Figure 3d). These findings support a role for SufB and PflA in *E. faecium* E980 Fe-S cluster biosynthesis and modification.

To explore which possible pathways downstream were affected by impaired Suf system, Fe-S cluster-containing proteins in E980 strain were identified by bioinformatical analysis. A total of 45 Fe-S cluster-containing proteins in *E. faecium* were identified through homology comparison with *E. coli* Fe-S cluster proteins and retrieval from UniProt (Table S4). Subsequently, these 45 downstream proteins were further compared to E980 genome, resulting in a final set of 26 proteins. These 26 proteins are involved in various pathways, including biosynthesis; DNA repair; Fe, S, and N metabolism; gene regulation, post-translational modification; respiration; RNA modification; and other crucial cellular processes, as depicted in Figure 3e. We note that many known essential Fe-S cluster-modified proteins in *E. coli* have not been identified in E980, such as aconitate hydratase (AcnA) and fumarate hydratase (FumAB). Due to the limitations of this methodology, Fe-S cluster-containing proteins in E980 strain were not fully identified. Even so, these findings suggest that biosynthesis pathway of Fe-S cluster plays a pivotal role in diverse bacterial physiological processes.

The functional analysis of *suf* gene cluster in *E. faecium* support a role for SufB and PflA in *E. faecium* E980 Fe-S cluster biosynthesis and modification, as well as diverse bacterial physiological processes.

### 3.5. Growth of *E.* *faecium* under aerobic and anaerobic conditions

A growth curve was generated for WT, Δ*sufB::gm*, and Δ*sufB::gm* + *sufB* strains under both aerobic (Figure 3f) and anaerobic (Figure 3g) conditions. Similarly, growth curves of WT, Δ*pflA::gm*, and Δ*pflA::gm* + *pflA* strains were generated under both aerobic (Figure 3h) and anaerobic (Figure 3i) conditions. Both WT and isogenic mutants exhibited indistinguishable growth patterns under aerobic conditions, suggesting that the introduced mutations did not exert any discernible impact on aerobic growth. However, deletion of *sufB* and *pflA* genes had a significant impact on anaerobic growth of *E. faecium* E980. The difference between Δ*pflA::gm* strain and WT strain was only statistically significant at the fourth hour. The results indicate that *sufB* and *pflA* genes of *E. faecium* play crucial roles in E980 anaerobic growth.

### 3.6. Transcriptome comparison between WT and Δ*sufB::gm* strains under aerobic and anaerobic conditions

**Figure 4.**
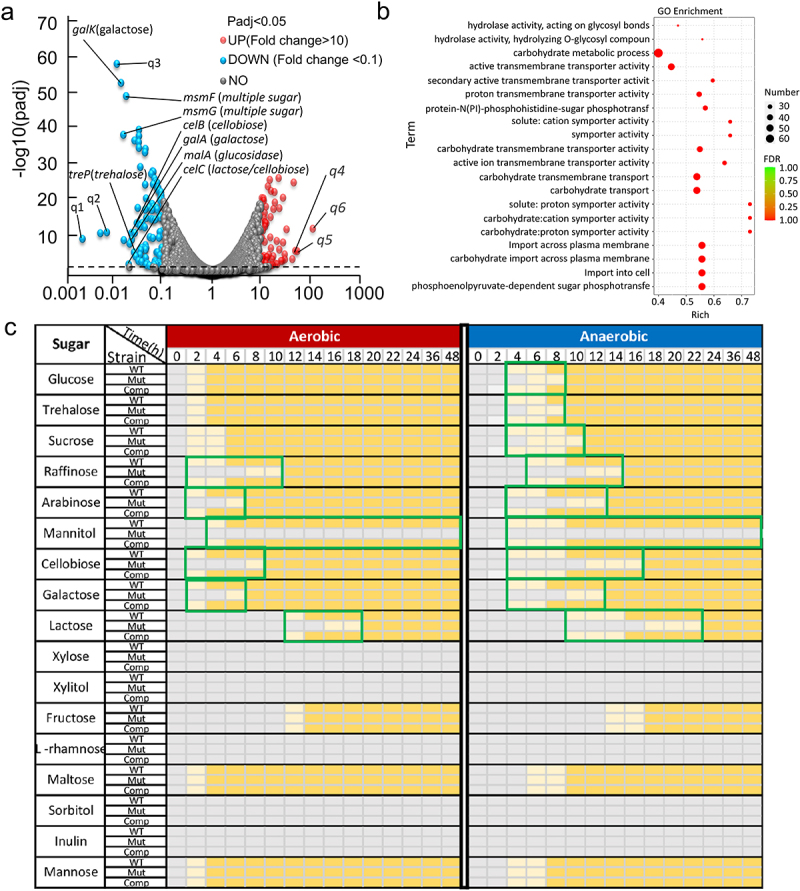
Transcriptome comparison between WT and *sufB* mutant strains under anaerobic conditions.

Transcriptomic analysis was also conducted to investigate the differences in gene expression between WT and Δ*sufB::gm* strains during anaerobic growth. Significant disparities in the heatmap (Figure S4) between two groups indicate notable variations in expression patterns between WT and Δ*sufB::gm* under anaerobic conditions. Further analysis of the data revealed 1099 DEGs for genes with a P_adj_ value < .05 and a fold change > 2. Among the 1099 genes, 552 genes were upregulated, whereas 547 genes were downregulated (Table S6). Moreover, when we further increased the fold change requirement (FC > 10 or FC < 0.1), there were still 54 genes were upregulated, whereas 101 genes were downregulated (Figure 4a). Deletion of *sufB* resulted in a large proportion of DEGs under anaerobic conditions. GO enrichment analysis was subsequently performed to determine the functions of DEGs. The most significant top 20 GO terms of downregulated DEGs are shown in Figure 4b. Out of the total of 20 terms, 7 terms were associated with carbohydrate metabolism. The 7 terms included carbohydrate metabolic process, carbohydrate transmembrane transporter activity, carbohydrate transmembrane transport, carbohydrate transport, carbohydrate: cation symporter activity, carbohydrate: proton symporter activity, and carbohydrate import across plasma membrane. This result may indicate that deletion of *sufB* gene in *E. faecium* affects carbohydrate metabolism. Furthermore, the real-time PCR results confirmed reliability of RNA-Seq analysis, as shown in Figure S6.

(a) Bubble plot of the DEGs (WT vs Δ*sufB::gm*). The dashed line indicates that *P*-value is equal to .05. The highly down-regulated and carbohydrate-related genes were labeled, in which the corresponding sugars are indicated in parentheses; q1-q6 represent the genes that were used for qPCR validation (Figure S6). (b) The top 20 enriched GO terms of the DEGs. (c) Bacterial strains (WT, Δ*sufB::gm* and Δ*sufB::gm* + *sufB*) were tested for their ability to metabolize 17 different sugars under aerobic and anaerobic conditions. The color gray and dark yellow represent negative and positive results for sugar metabolism, respectively. The light yellow represents intermediate result. The periods of metabolic gap between mutant and WT are marked in green boxes.

In order to study the influence of *sufB* deletion in *E. faecium*, we conducted an RNA-Seq experiments to illustrate the transcriptional effect of *sufB* deletion on *suf* gene cluster in different conditions (TableS5-S8). Under aerobic conditions, O_2_ can damage the homeostatic state of Fe-S cluster, so the demand for Fe-S cluster is higher, which is also verified by the RNA-Seq results in this study. Under aerobic conditions, the impact of *sufB* deletion on Fe-S cluster generation may be small (Figure 3f), which is also illustrated by RNA-Seq: even if there is a high demand for Fe-S cluster under aerobic conditions, *sufB* deletion does not up-regulate the expression of *sufCDSU* gene.

### 3.7. Deletion of *sufB* results in extensive and significant impairments on carbohydrate metabolism

We have conducted a metabolic experiment on 17 different sugars. In general, all strains (WT, *sufB* mutant, and complement strain) grow faster in aerobic conditions compared to anaerobic conditions (Figure 4c). In aerobic conditions, although deletion of *sufB* does not affect the growth on glucose and several other sugars, the growth on raffinose, *etc*. especially mannitol is significantly suppressed, indicating that the essentiality of *sufB* depends on the particular carbon source. Under anaerobic conditions, the utilization of those unaffected sugars (including glucose, trehalose and sucrose) in aerobic conditions are impaired, suggesting that deletion of *sufB* under anaerobic conditions results in more extensive and significant impairments on carbohydrate metabolism compared to aerobic conditions. Interestingly, the type of “slow-growth carbohydrates” of *sufB* mutant were highly correlated with the function of down-regulated genes in transcriptome analysis as shown Figure 4a,c. The results established a link between phenotype and gene regulation, further revealing the mechanism by which *sufB* affects bacterial growth under anaerobic conditions.

### 3.8. *sufB* and *pflA* are required for colonization of intestinal tract by *E.* *faecium*

**Figure 5.**
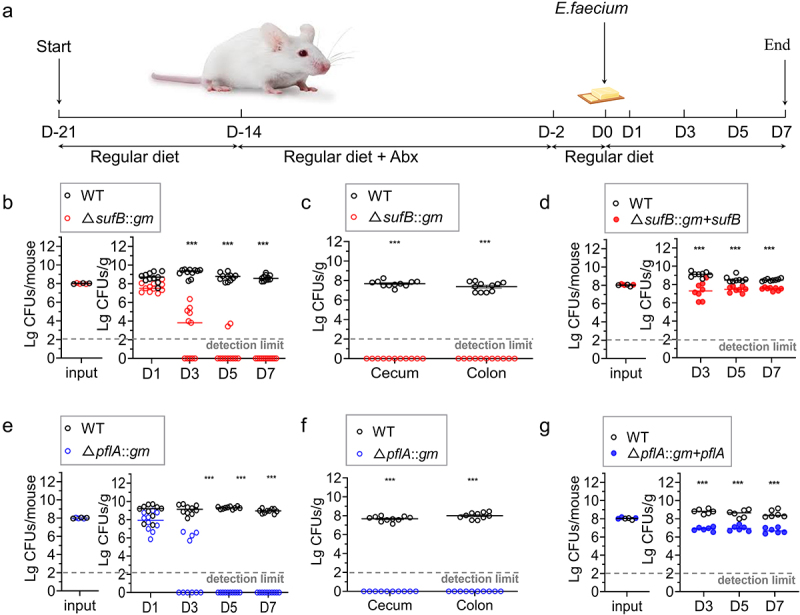
Colonization of intestine by *E. faecium* in a mouse model.

To further investigate the impact of *sufB and pflA* of *E. faecium* on intestinal colonization in mice, cell suspensions of E980 strain and Δ*sufB::gm* were mixed in a 1:1 ratio (10^8^ CFUs of each strain) and administered to mice via oral infection (Figure 5a). Similarly, the other mutant, Δ*pflA::gm*, underwent identical treatment procedures. As shown in Figure 5b,e. *E. faecium* colonized intestinal tract of mice at high levels. There was a constant colonization rate, as the concentration in feces samples was always >10^9^ CFUs/g. In competitive colonization assays, based on all stool samples collected across all time points, results showed that mutant isolate had significantly fewer viable cells compared to WT strain, as shown in Figure 5b,e. Mutant became almost undetectable by 3 days after infection. A completely lower level of colonization in cecum and colon was observed with mutant, as shown in Figure 5c,f, compared to parental strain. Cell suspensions of E980 strain and Δ*sufB::gm* + *sufB* were mixed in a 1:1 ratio (10^8^ CFUs of each strain) and administered to mice via oral infection. As shown in Figure 5d,g, it can also be proved *E. faecium* colonized intestinal tract of mice at high levels. There was a constant colonization rate, as the concentration in feces samples was always >10^9^ CFUs/g. Although the number of Δ*sufB::gm* + *sufB* is not as much as WT, it is far more than the number of Δ*sufB::gm*. Results showed that although it could not be fully complemented, complementary strain could rescue colonization to a fairly level which is significantly higher than mutants. Incomplete complementarity may be due to instability of plasmid *in vivo* environment or excessive copy number of plasmid, gene expression disorder and other predictable and common reasons. This result further confirms our previous findings.

(a) Flow chart of animal experiments. Ten mice were inoculated with pure cultures in competition experiments with 1:1 mixtures of *sufB* mutant and WT. Samples of feces were collected on Days 1, 3, 5, and 7 after colonization, and samples of the cecum and colon were collected on Day 7 of colonization. (b) and (c), The numbers of *E. faecium* in feces, cecum, and colon of WT and Δ*sufB::gm* strains were determined. respectively. The other mutant, Δ*pflA::gm*, underwent the same treatment (e) and (f). Mice were inoculated with pure cultures in competition experiments with 1:1 mixtures of Δ*sufB::gm* + *sufB* and WT. Samples of feces were collected on Days 3, 5, and 7 after colonization. (d) The numbers of *E. faecium* in feces of WT and Δ*sufB::gm* + *sufB* strains were determined. respectively. The other strain, Δ*pflA::gm* + *pflA*, underwent the same treatment. (g) The data are presented in the form of a scatter plot, displaying the median. The *P* values obtained through paired 2-tailed Student’s t tests are indicated for the comparisons between WT and mutant strains. CFU is an abbreviation for colony-forming units. ***P < .001. *n* = 10 in each group.

Based on these results, we concluded that deleting *sufB and pflA* in *E. faecium* E980 resulted in diminished intestinal colonization in mice.

## 4. Discussion

The majority of current research on *Enterococcus* has been conducted under aerobic conditions, with limited attention given to its physiological responses in anaerobic environments. The mechanisms by which *Enterococcus* maintain proliferation under anaerobic conditions are poorly understood, resulting in a significant knowledge gap. Therefore, it is important to explore the growth mechanism of *E. faecium* under anaerobic conditions. This study provided a comprehensive genome-wide identification of essential genetic determinants under anaerobic conditions in *E. faecium*.

In this study, Tn-seq methodology was used to elucidate genetic determinants involved in anaerobic growth. *E. faecium*, as a gut commensal, must have developed mechanisms to sense, respond to, and adapt to anaerobic environments.^52^ Deletion of *sufB* and *pflA* genes had a significant impact on anaerobic growth of *E. faecium* E980 but had no discernible influence on its aerobic growth (Figure 3g,i), which verified the results of Tn-seq analysis. The role of *sufB* and *pflA* in gut colonization was demonstrated through competition experiments with WT and isogenic mutants. Our study emphasizes the significance of Fe-S cluster modification system in facilitating the proliferation of *E. faecium* under anaerobic conditions and gastrointestinal colonization.

The *suf* gene cluster was found to be widely spread across diverse bacterial species (Figure 3a). Fe – S cluster exhibited a nearly ubiquitous presence in both aerobic and anaerobic Archaea, Bacteria, and Eukarya.^50^ Fe – S cluster are highly conserved cofactors in both prokaryotes and eukaryotes.^59^ Suf system, an upstream pathway of Fe-S cluster biosynthesis, regulates numerous functional Fe – S cluster proteins and has prominent roles in multiple important cellular processes,^60^ including respiration, central metabolism, gene regulation, RNA modification, DNA repair and replication. *E. coli* contains approximately 140 Fe-S proteins.^61^ In this study, a total of 26 putative functional Fe – S cluster proteins were identified in *E. faecium* E980. Further research on *E. faecium* is anticipated to reveal additional functional Fe – S cluster proteins, particularly those associated with sugar metabolism, which could provide novel insights into carbohydrate-dependent mechanisms that contribute to intestinal colonization of gut microbes.

In order to find out whether *E. faecium* encodes any other Fe-S cluster biogenesis systems than Suf pathway, we performed bioinformatical analyses of Isc, Nif and recently identified MIS (minimal iron-sulfur) and SMS (SUF-like minimal system) based on protein sequence (blastp) and structure (HHpred), and confirmed that none of systems is present in *E. faecium*.^62^ Therefore, results show that Suf system is only known Fe-S cluster biogenesis system in *E. faecium*. Our results of essential gene analysis showed that *sufB* is not essential while *sufU* is essential for *E. faecium*. The similar results were also observed in *B. subtilis* and *S. aureus*, in which the disruption of *sufB, sufC, sufD* or *sufS* was not lethal.^63–65^ Therefore, we believe that although Suf system is necessary for bacterial viability, not every *suf* gene is required. The results suggest that the dependence of Fe-S cluster on *sufB* is different under aerobic and anaerobic conditions. Compared with aerobic conditions, the formation of effective Fe-S cluster under anaerobic conditions is more dependent on an intact (all genes of *sufCDSUB* cluster) *suf* gene cluster.

A transcriptome comparison between WT and *sufB* mutant strains demonstrated that *suf* deficiency resulted in a large proportion of DEGs under anaerobic conditions (Figure 4b), and there was notable enrichment in pathways related to sugar metabolism (Figure 4c). The *suf*-encoded Fe-S cluster biosynthesis pathway is an important means of protein modification, in which Fe-S cluster are modified into numerous proteins to regulate their function.^57^ We hypothesized that proteins encoded by these genes involved in glycometabolism may exhibit impaired functionality due to deficient Fe-S cluster modification, thereby resulting in attenuated growth of *suf*-deficient mutant under anaerobic conditions (Figure 3g).

Bacteria use different energy metabolism pathways under aerobic and anaerobic conditions.^66^ Under anaerobic conditions, bacteria do not have exogenous electron acceptors and cannot achieve complete tricarboxylic acid cycle (TCA cycle), and mainly perform fermentation to release energy lower compared to aerobic conditions. The formula was: glucose→2 pyruvate→2 acetate + ethanol + formate + lactate + 2 CO_2_. During this process, pyruvate is converted into acetyl-CoA and formate by pyruvate formate lyase (PflA), which is then metabolized to CO_2_ and H_2_ by formate dehydrogenase (Fdh).^67^ Both PflA and Fdh are Fe-S cluster modified enzymes.^68,69^ Under anaerobic conditions, the restriction of Fe-S cluster generation caused by *sufB* deletion prevents PflA and Fdh from being modified by Fe-S cluster and thus cannot exert catalytic activity, which reduces the efficiency of process. In this study, the importance of *pflA* gene on anaerobic growth and colonization was also screened by Tn-seq, and verified by *in vitro* and *in vivo* experiments (Figures 1c, 3 i and 5e-g), which further confirmed the important role of Fe-S cluster modification on anaerobic respiration. We performed a further RNA-Seq experiment to check gene regulation of WT *E. faecium* under aerobic and anaerobic conditions. As expected, the pathways relevant to pyruvate metabolism and TCA cycle were significantly down-regulated (Figure S5 and Table S7).

Deletion of *sufB* results in more extensive and significant impairments on carbohydrate metabolism under anaerobic conditions compared to aerobic conditions. Results are consistent with the growth experiment on BHI (Figure 3f,g) in which the main carbon source is glucose. Mice were fed with commercial mice feed in which starch is the main carbon source. We attempted to grow *E. faecium* on the medium made with mice feed, however, probably because *E. faecium* cannot utilize starch directly as carbon source, Cell density could only reach to approximately 10^7^, which is too low to perform growth experiment. However, in the gut starch could eventually be digested to glucose by the host and gut microbes. Therefore, the relative inefficiency of glucose metabolism of *sufB* mutant under anaerobic conditions could be one of factors that leads to the failure of intestinal colonization.

Our study revealed the pivotal role of Fe-S cluster biosynthesis system in intestinal colonization of *E. faecium*, thereby providing compelling evidence for investigating the key determinants of gut microbial colonization.

## Supplementary Material

Supplemental Material

## Data Availability

The datasets generated during this study are available in the National Center for Biotechnology Information (NCBI) BioProject Repository https://www.ncbi.nlm.nih.gov/bioproject under BioProject PRJNA1061835.
