# Supplementary material for: The Fe-S cluster biosynthesis in Enterococcus faecium is essential for anaerobic growth and gastrointestinal colonization
Source: Gut Microbes. 2024 Jun 3;16(1):2359665. doi: 10.1080/19490976.2024.2359665 (PMC11152105; doi:10.1080/19490976.2024.2359665)
Supplement: Supplemental Material [file KGMI_A_2359665_SM2909.zip › Table S1 .docx]

# Table S1. Strains, plasmids, and primers.

| Strains, plasmid, or primer | Relevant features (Primer sequence 5'-3') |
| --- | --- |
| Strains |  |
| *E.faecium* E980 | Genome is sequenced |
| *E. coli* EC1000 | Cloning host strain |
| Δ*sufB*::*gm* | E980Δ*sufB*::*gm* |
| Δ*pflA*::*gm* | E980Δ*pflA*::*gm* |
| Plasmids |  |
| pGPA2 | mariner delivery vector; Cm r ; *Gm* r |
| pWS3 | Temperature-sensitive integration vector; Spc r |
| Primers |  |
| pAT392_lox66_genta_F | TACCGTTCGTATAGCATACATTATACGAAGTTATGATAAACCCAGCGAACCATTTGAGG |
| pAT392_lox71_genta_R | TACCGTTCGTATAATGTATGCTATACGAAGTTATTCAATCTTTATAAGTCCTTTTATAA |
| genta2_F | ACCTCAAGTACCGTTCGTATAGCATACA |
| genta2_R | GTGTAGCTTACCGTTCGTATAATGTATG |
| Genta2_1_R | TCAAGGCAATCTGCCTCCTCA |
| Genta2_2_F | ATGGAAAGACTAAATGCAACAACA |
| pWS3_*gm*_ud_F1 | GGGGATTTTATGCGTGAGAA |
| pWS3_*gm*_ud_R1 | GCTTCCAAGGAGCTAAAGAGG |
| *sufB*_up_F | AAGCTGAATTCCTGCAGCCCTCTTCCCATCCACATCATCGT |
| *sufB*_up_R | TGTATGCTATACGAACGGTACTTGAGGTTCTGTCAAGCCTTCTCCTGT |
| *sufB*_down_F | GCATACATTATACGAACGGTAAGCTACACGCCTGACCGAATCTGAAGCA |
| *sufB*_down_ R | CTAGAACTAGTGGATCCCCCAGCTCAGCCTTCGCAAGTTT |
| Check_*sufB*-F | TCTTCCCATCCACATCATCGT |
| Check_*sufB*-R | AGCTCAGCCTTCGCAAGTTT |
| *pflA*_up_F | AAGCTGAATTCCTGCAGCCCCATCCAACTGGTTCTGCACG |
| *pflA*_up_R | TGTATGCTATACGAACGGTACTTGAGGTTTGGAAGGAATCGAACCACCT |
| *pflA*_down_F | GCATACATTATACGAACGGTAAGCTACACAAACACGACAAAGCGCAGTC |
| *pflA*_down_ R | CTAGAACTAGTGGATCCCCCCGGATACTTCTCAAAAGGGGCT |
| Check_*pflA*-F | CTGAAAGCATGATCCCAGCAA- |
| Check_*pflA*-R | CGGATACTTCTCAAAAGGGGC |
