# Supplementary material for: The Fe-S cluster biosynthesis in Enterococcus faecium is essential for anaerobic growth and gastrointestinal colonization
Source: Gut Microbes. 2024 Jun 3;16(1):2359665. doi: 10.1080/19490976.2024.2359665 (PMC11152105; doi:10.1080/19490976.2024.2359665)
Supplement: Supplemental Material [file KGMI_A_2359665_SM2909.zip › Table S5 .docx]

Table. S5 Transcriptome comparison of *suf* gene cluster between WT and *sufB* mutant under aerobic and anaerobic conditions.

| Gene | Anaerobic-WT VS Aerobic-WT | | Aerobic -Δ*sufB*::*gm* VS Aerobic -WT | | Anaerobic -Δ*sufB*::*gm* VS Anaerobic -WT | |
| --- | --- | --- | --- | --- | --- | --- |
|  | Log_2_FoldChange | Pval | Log_2_FoldChange | Pval | Log_2_FoldChange | Pval |
| *sufC* | -1.60093744 ↓ | 1.617E-05 | 0.573649807 **—** | 0.0765861 | 1.433171↑ | 5.29E-05 |
| *sufD* | -1.600132526↓ | 3.420E-05 | 0.403953507 **—** | 0.2311479 | 1.817265↑ | 5.66E-07 |
| *sufS* | -1.582872859↓ | 2.134E-05 | -0.214734004 **—** | 0.4967298 | 2.172488↑ | 1.06E-06 |
| *sufU* | -1.854617154↓ | 4.735E-05 | 0.036948388 **—** | 0.9545222 | 1.366196↑ | 0.002018 |
| *sufB* | -1.47791711 ↓ | 1.895E-05 | -8.171004526↓ | 1.434E-62 | -7.6612 ↓ | 1.8E-12 |

↑Up-regulated ↓Down-regulated **—** No significant different
