# Supplementary figures and images for: The Fe-S cluster biosynthesis in Enterococcus faecium is essential for anaerobic growth and gastrointestinal colonization
Source: Gut Microbes. 2024 Jun 3;16(1):2359665. doi: 10.1080/19490976.2024.2359665 (PMC11152105; doi:10.1080/19490976.2024.2359665)

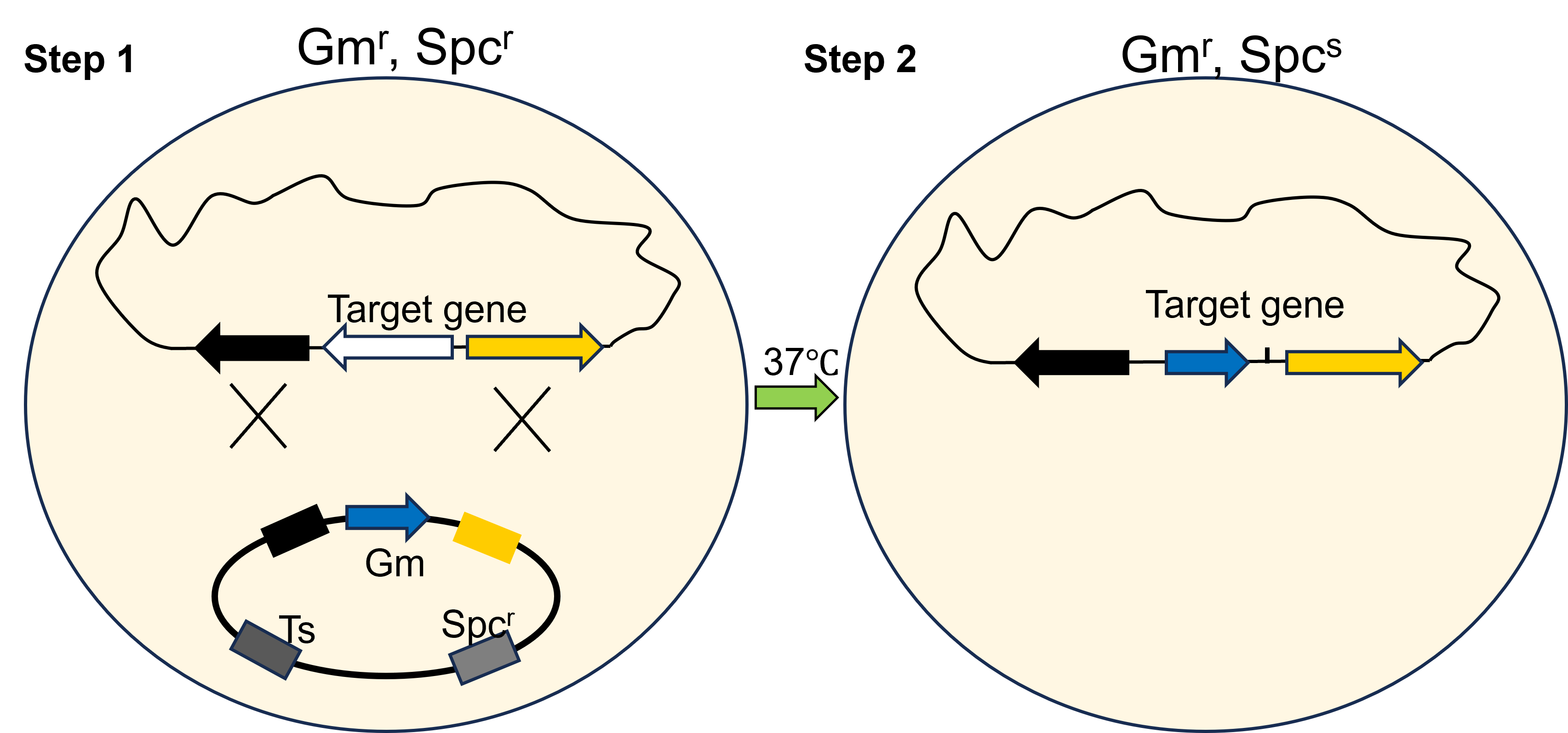

Supplement: Supplemental Material [file KGMI_A_2359665_SM2909.zip › Figure S1.tif]

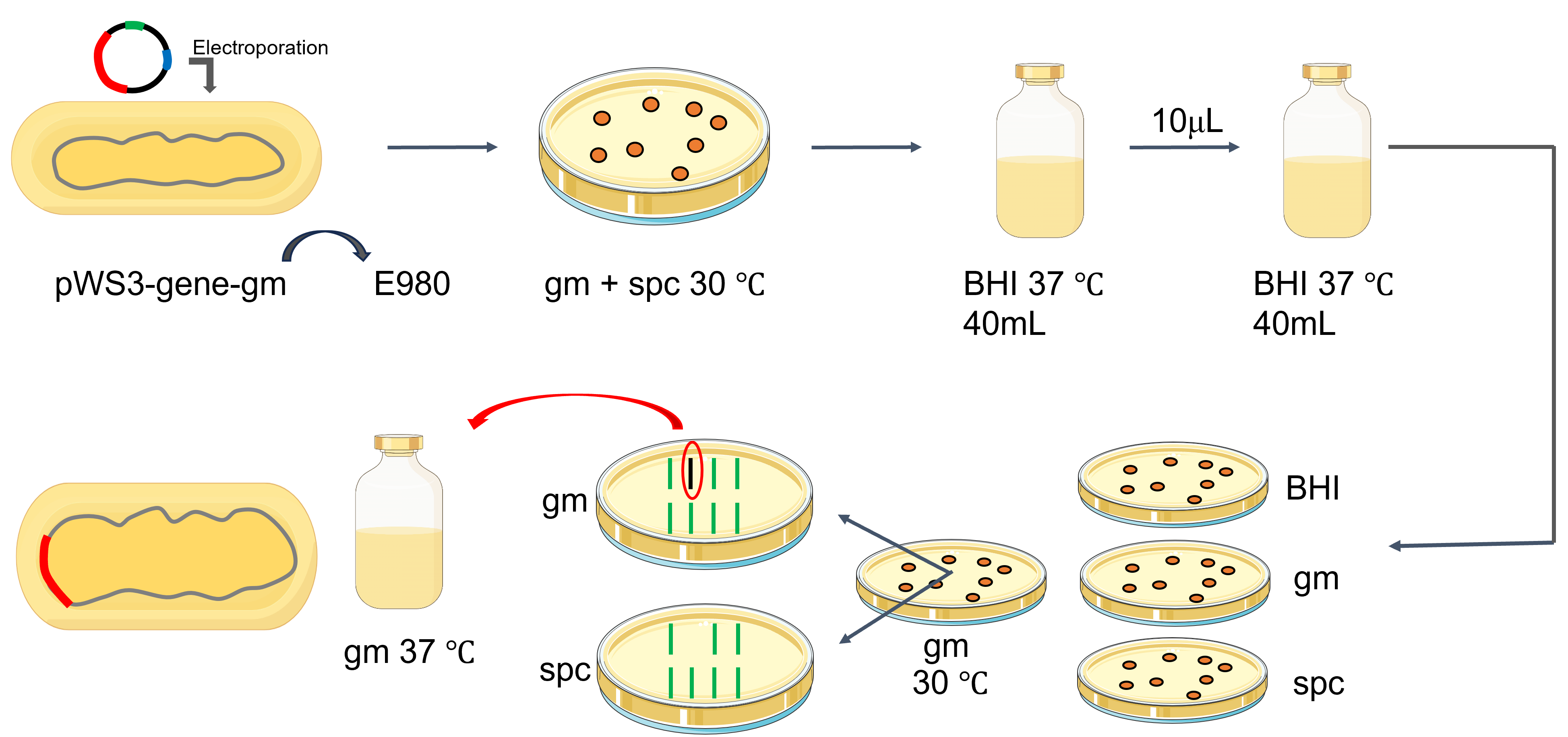

Supplement: Supplemental Material [file KGMI_A_2359665_SM2909.zip › Figure S2.tif]

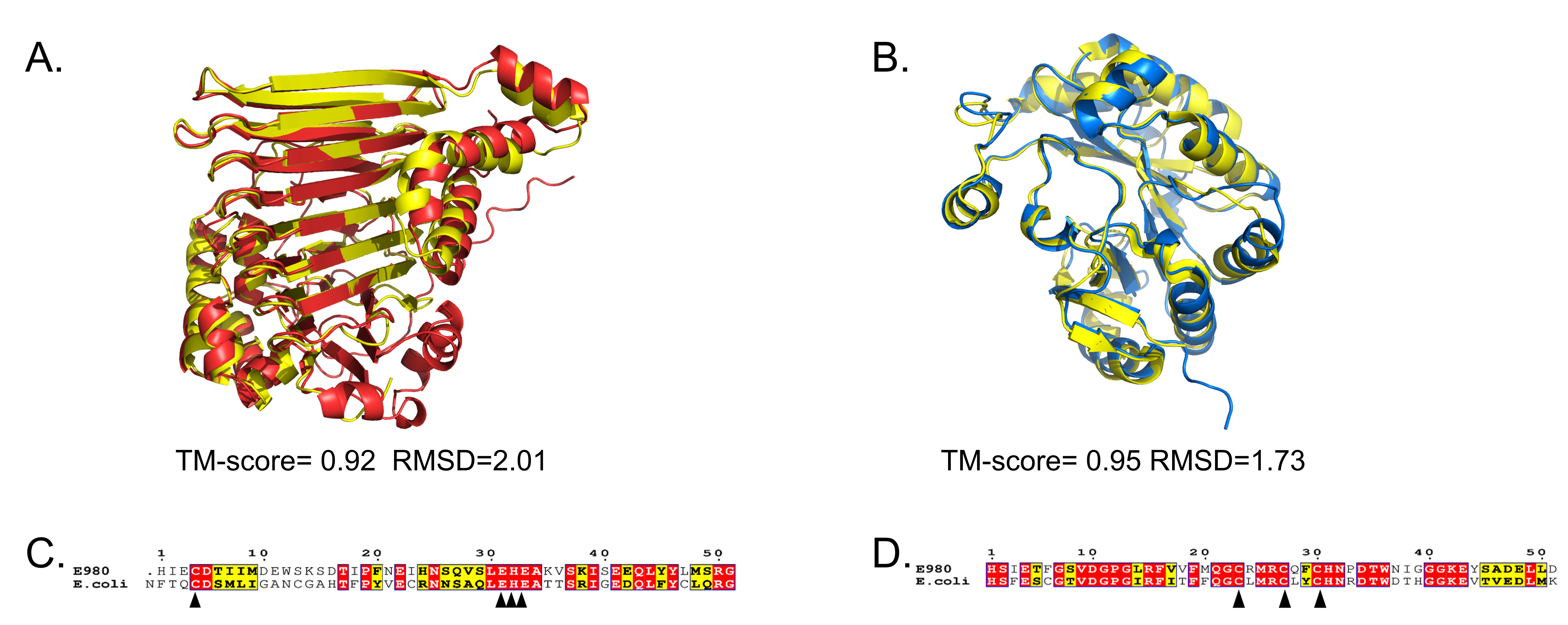

Supplement: Supplemental Material [file KGMI_A_2359665_SM2909.zip › Figure S3.tif]

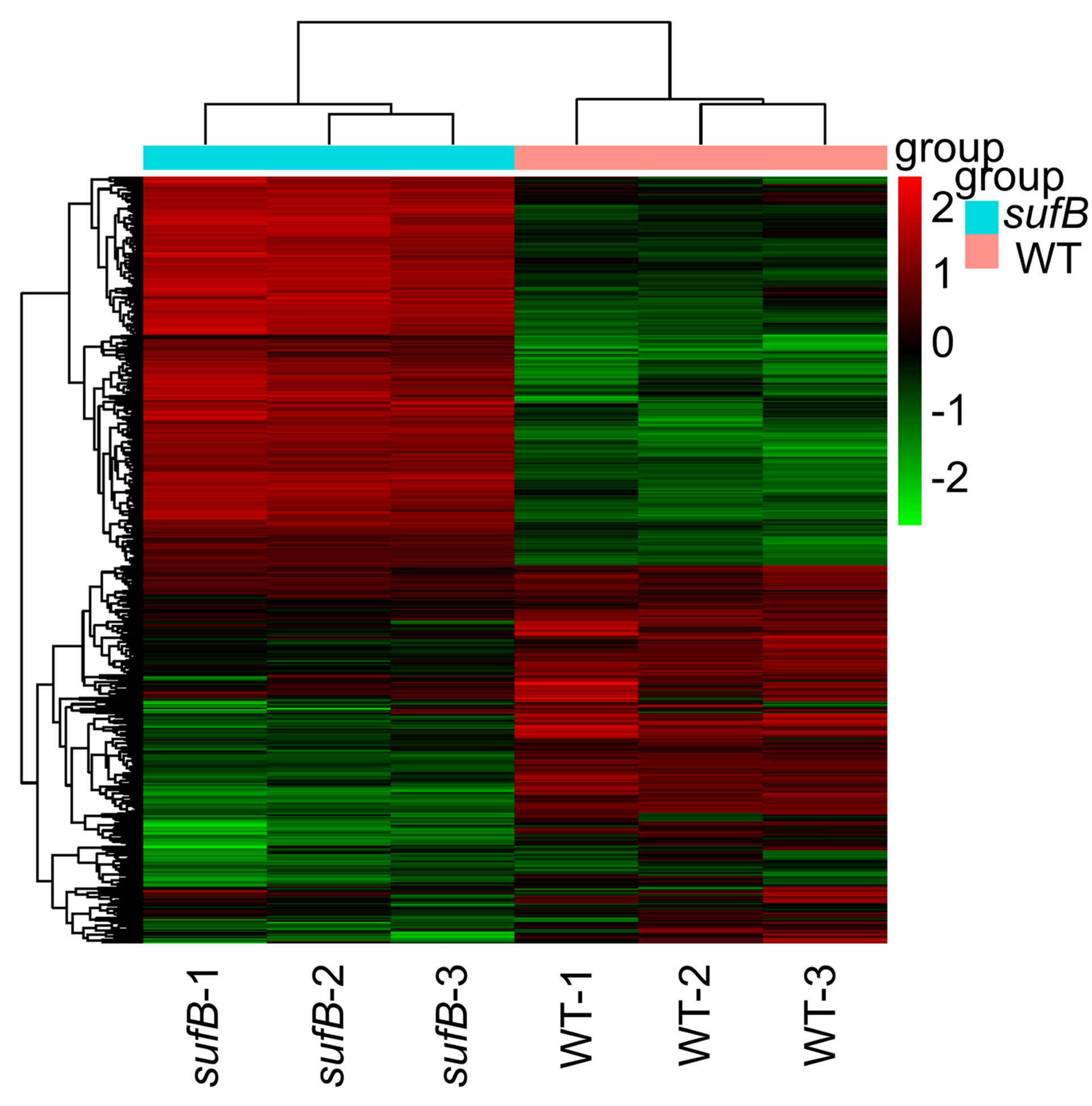

Supplement: Supplemental Material [file KGMI_A_2359665_SM2909.zip › Figure S4.tif]

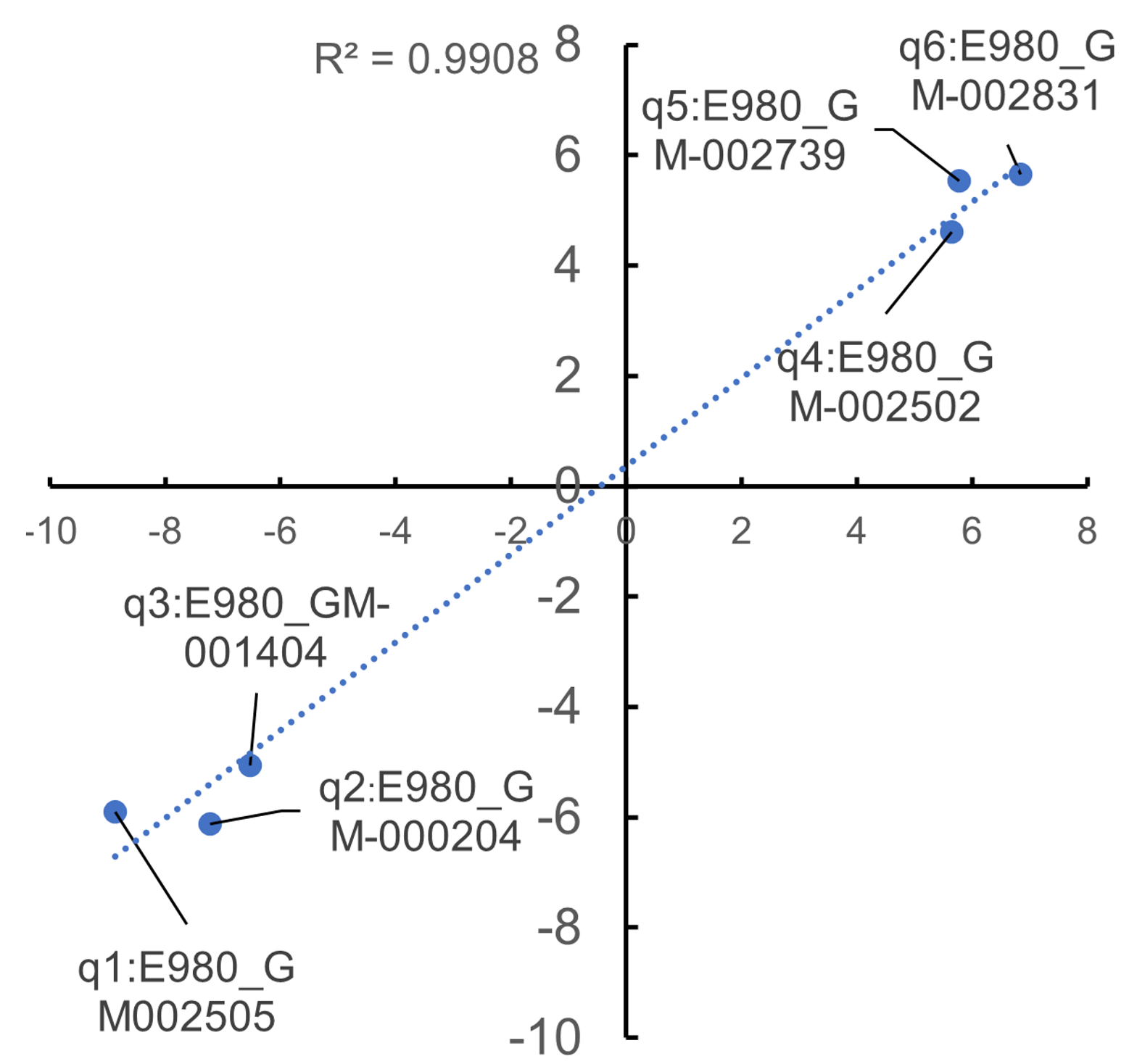

Supplement: Supplemental Material [file KGMI_A_2359665_SM2909.zip › Figure S6.tif]
